# Vitamin D: An overview

**DOI:** 10.31138/mjr.28.4.206

**Published:** 2017-12-22

**Authors:** Chris Clifopoulos

**Affiliations:** School of Medicine, The University of Queensland, Australia

**Keywords:** Cholecalciferol (vitamin D3), Ergocalciferol (vitamin D2), 25-hydroxy vitamin D (25 OHD), basal cell carcinoma (BCC)

## Abstract

The role of Vitamin D is examined in skin with regards to synthesis and neoplasia. Despite its evolving role in bone health undefined benefits with regards to other disease such as neoplasia and skin cancer are under scrutiny. A brief overview is presented with formalised advice to patients regarding skin cancer, and Vitamin D requirements in the clinical setting. Given that weather conditions in Australia and the Mediterranean climates share similarities, some parallels can be drawn from the Australian experience for Europe.

## INTRODUCTION

Vitamin D is an ancient hormone produced in plants and animals having evolved over a long evolution period.^1^ Shrimp and krill have been found to contain Vitamin D precursors (ergosterol and 7 dehydrocholesterol) which is converted by ultraviolet B (UVB) radiation to calcium exoskeletons in higher marine mammals and sea life. The best and most noted effects of bioactive Vitamin D 1,25(OH)2D is found once it is converted to active form in sun exposed tissues, and naturally, this commences in the skin and, finally, the kidney. Calcium homeostasis is the end effect of the bioactive form using genomic and non-genomic effects through the Vitamin D receptor in cells and activation of other physiological effects. These have been postulated to be apart from bone health and calcium homeostasis, immune regulation and prevention of a variety of cancers.^1^

The relationship between skin cancer and diet has been investigated in a limited number of studies.

## PHYSIOLOGY

With reference to the skin, a model of Vitamin D and calcium homeostasis controlling keratinocyte differentiation has been proposed independent of bone mineralisation. The wavelength of UV radiation that causes DNA damage in skin cells (280–320 nM) also induces vitamin D production in keratinocytes. It is known that UV radiation from sun exposure is a main environmental risk factor for non-melanoma and melanoma skin cancer, which promotes differentiation and reduces proliferation in normal skin cells and skin cancer cell lines.^2^ Cholecalciferol (vitamin D3) is formed by action of UVB on 7-dehydrocholesterol in the skin. Most vitamin D supplements contain cholecalciferol. Ergocalciferol (vitamin D2) formed by UV radiation of the plant sterol ergosterol. Both forms of vita-min D are metabolised in the liver to 25-hydroxy vitamin D (25 OHD). This is the metabolite which is measured. 25 OHD is hydroxylated in the kidney to form the biologically active metabolite: calcitriol.

Calcitriol promotes absorption of calcium and phosphate from the gut. Both directly act through interaction with PTH, calcitriol controls extracellular calcium and phosphate homeostasis, facilitates mineralisation of the skeleton and influences muscle function.

## VITAMIN D LEVELS DISEASE AND MORTALITY

Diseases shown to be associated with low vitamin D: Rickets in children and osteomalacia in adults, autoimmune diseases, cardiovascular and metabolic diseases, some cancers, microbial and respiratory diseases, some neurological and mental health conditions including schizophrenia, all-cause and cardiovascular mortality.

In a 13 year follow up of 5,409 men (in a UK based prospective study), plus a meta-analysis of similar studies, and after adjusting for prior disease and cardiovascular risk factors, throughout the 40–90 nmol/L range, a doubling of 25(OH)D concentration was associated with 20% lower vascular mortality and 23% lower non-vascular mortality.^3^

There was no evidence that low vitamin D caused either vascular or non-vascular mortality.

There is no evidence to date that these conditions are caused by Vitamin D deficiency. It is possible that they both result from the same (e.g., lifestyle) factors.

## STUDIES ON THE IMPACT OF VITAMIN D SUPPLEMENTATION

No large randomised trials have been completed with vascular disease or cancer as the primary outcome.

Previous meta-analyses have reported only borderline statistically significant effects on all-cause mortality, with no detected effects on cardiovascular mortality.^3^

Moderated 25(OH) D deficiency is an independent predictor of falls (reduced by levels over 60 nmol/l and optimal mineralisation and muscle function is achieved at levels 50–60).

There is level 1 evidence re fall/fracture reduction: Vitamin D dose >800IU needs to be combined with calcium >1000 mg/day.

## VITAMIN D LEVELS AND SKIN CANCER RISK

The role of vitamin D in relation to skin cancer prevention has been the focus of substantial debate in recent years. It should be noted that cutaneous synthesis of vitamin D is self-limited and in light-skinned people it fades away after 5 to 10 minutes of sun exposure. Longer durations will not further increase vitamin D, but will increase skin cancer risk.

In a topical Queensland based prospective study,^4^ 1,191 adults were followed up after baseline 25(OH) D levels. After adjusting for time outdoors, age, gender and skin type, a level above 75nmol/L was associated with increased BCC risk (OR 1.51) (statistically significant) and melanoma risk (OR 2.71) (statistically borderline) and a decreased risk of SCC (OR 0.67) (not statistically significant).

There was no association of 25(OH) D levels and skin cancer risk above or below 50 nmol/L.

There is no evidence that elevated levels of 25(OH) D are protective against skin malignancy and high sun exposure is to be avoided as a means to achieve high vitamin D status.

By contrast a prospective European cohort study^5^ tested the hypothesis that elevated plasma 25-hydroxyvitamin D (25-OH-vitD) associates with increased risk of nonmelanoma and melanoma skin cancer in the general population. Plasma 25-OH-vitD levels were measured in 10,060 white individuals from the Danish general population. The absolute 20-year risk was 11% for nonmelanoma skin cancer and 1.5% for melanoma skin cancer, in participants with age 460 years, 25-OH-vitD winter levels X50 nmol l_1, and performing outdoor exercise. In conclusion, we show that increasing levels of 25-OH-vitD are associated with increased risk of non-melanoma and melanoma skin cancer.

In another case control study,^6^ there was a significant decreased risk of melanoma associated with a high dietary intake of Vitamin D (also for Vitamin A) the odds ratio for Vitamin D being 0.61 at the highest level measured, this being a linear dose-dependent relationship. It was not increased by oral supplementation compared to dietary vitamin D.

A comprehensive meta-analysis^7^ suggests a possible significant role of VDR FokI and BsmI polymorphism in cutaneous melanoma and non-melanoma skin cancer risk. The association with Vitamin D intake was less clear and further studies could be useful to clarify the role of diet.

In summary, a diet rich in vitamin D was shown to reduce melanoma risk almost by half but supplementation with non-food vitamin D had no additional impact in this study. The study was looking at vitamin D in the diet and not at vitamin D levels in the blood.^4^

## FACTORS WHICH INFLUENCE VITAMIN D LEVELS

- UVB radiation which by far is also the biggest confounder in studies to date
- Dietary sources account for 5–10% of vitamin-d. Fatty fish (salmon, herring, mackerel), liver, eggs and fortified food are known to provide high levels of VitD.
- Vitamin D supplements
- Obesity: Associated with lower vitamin D levels including after supplementation (vitamin D enters adi-pose tissue and becomes unavailable)
- Medication: Some medications accelerate degradation of vitamin D compounds (Cytochrome p450 enzyme inducers and some anticoagulants)
- Low calcium intake: Leads to accelerated 25 OHD degradation

**Table 1.** Baseline characteristics according to clinical categories for 25-hydroxyvitamin D plasma levels *Afzal et al., J Invest Dermatol. 2013 ;133:629–36 - reproduced with permission5

	**Plasma 25-hydroxyvitamin D (nmoll^−1^**)				**Non-melanoma skin cancer**		**Melanoma**	
	<**25 (*n* = 2,362)**	**25–49.9 (*n* = 4,035)**	>**50 (*n* = 3,663)**	***P*-value^1^**	**HR (95% CI)**	***P*-value^2^**	**HR (95% CI)**	***P*-value^2^**
Men, no. (%)	1,074 (45)	1,760 (44)	1,575 (43)	0.07	1.26 (1.07–1.49)	0.007	1.47 (0.94–2.30)	0.09
*Age, years*				<0.001		<0.001		0.13
Median	59	59	57					
Interquartile range	50–65	49–65	48–65					
*Cumulative tobacco consumption, pack-years*				<0.001	1.00 (0.99–1.00)	0.84	0.99 (0.98–1.01)	0.82
Median	24	19	18					
Interquartile range	10–38	5–33	3–31					
*Body mass index, kg m^−2^*				<0.001	0.98 (0.96–1.00)	0.04	0.98 (0.93–1.04)	0.60
Median	25	25	24					
Interquartile range	23–29	23–28	22–27					
*Income group, no. (%)*^3^				<0.001		<0.001		0.55
Low (<84,000 DKr)	927 (40)	1,262 (32)	974 (27)		1		1	
Medium (84,000–192,000 DKr)	1,031 (44)	1,874 (47)	1,714 (47)		1.15 (0.93–1.43)		1.43 (0.77–2.66)	
High (>192,000 DKr)	368 (16)	844 (21)	941 (26)		1.57 (1.23–2.00)		1.28 (0.62–2.65)	
*Occupational physical exertion, no. (%)*				0.07^4^		0.06^4^		0.35^4^
Low	798 (34)	1,363 (34)	1,273 (35)		1		1	
Occasional	864 (37)	1,596 (40)	1,480 (40)		0.76 (0.63–0.92)		1.06 (0.61–1.84)	
Moderate	510 (22)	858 (21)	752 (21)		0.88 (0.70–1.10)		1.36 (0.75–2.50)	
High	108 (5)	141 (3)	111 (3)		0.68 (0.40–1.16)		1.23 (0.36–4.15)	
Without work	82 (3)	77 (2)	47 (1)		—		—	
*Leisure-time activities, hours per week*^2^				<0.001		0.08		0.03
⩽2	598 (25)	686 (17)	410 (11)		1		1	
2–4 (light activity)	1,115 (47)	1,986 (49)	1,792 (49)		1.14 (0.88–1.47)		0.89 (0.43–1.81)	
2–4 (heavy activity) or ⩾4 (light activity)	622 (26)	1,282 (32)	1,346 (37)		1.25 (0.96–1.63)		1.61 (0.96–1.63)	
⩾4 (heavy activity)	27 (1)	79 (2)	115 (3)		1.36 (0.75–2.46)		1.87 (0.50–7.03)	
*Regular cycling or running*				<0.001		<0.001		0.02
No	1,545 (65)	2,274 (56)	1,774 (48)		1		1	
Yes	817 (35)	1,761 (44)	1,889 (52)		1.36 (1.15–1.61)		1.75 (1.10–2.80)	

Abbreviations: CI, confidence interval; HR, hazard ratio.

1 *P*-values were calculated using Cuzick’s non-parametric trend test.

2 Trend test or use of continuous variable, Cox regression.

3 1981–1983 income levels (1 US$ ∼6 DKr).

4 Excluding participants not working. The following covariates had missing values: income group: 125 observations, leisure-time activities: 2 observations, and body mass index: 18 observations.

In a quantitative study on 1,002 Australian adults in 4 sites spanning 24 degrees latitude, 40% of the variance in 25(OH)D levels was determined by factors defined in the study and of this 40% the breakdown was:

- 52%: modifiable behavioural factors (clothing cover 27%, personal UVR exposure 8%, vitamin D supplementation 7%, physical activity 4%)
- 38% environmental factors (latitude 20%)
- 10% constitutional factors (including BMI)

The following were ***not*** significant determinants: Dietary intake of vitamin D-rich foods, skin type (the number of participants with dark skin was small) and sunscreen use.

## CLINICAL IMPLICATIONS AND RELEVANT INFORMATION FOR PATIENTS

1. There is evidence that the optimum range of 25(OH) D is between 50–75 nmol/L with respect to bone, muscle health and falls-prevention, and that vitamin D supplements are beneficial in both increasing 25(OH) D to these levels and in preventing falls and fractures.
2. A suitable dose for supplementation to prevent fracture risk in older people is 1000 IU per day combined with adequate calcium intake.
3. There is evidence that optimum levels as defined above are associated with a decrease in cardiovascular and all-cause mortality but as it is possible that lifestyle factors underlie both optimum levels and lower mortality; a lifestyle that results in optimum levels may be more important that actual supplementation.
4. Attempts to increase 25(OH) D levels by dietary modification are likely to produce only minimal changes.
5. A diet rich in vitamin D (but not vitamin D supplements), but not high blood levels of vitamin D, has been shown to reduce melanoma risk by almost half.
6. For moderately fair-skinned people, a walk with arms exposed for 6–7 minutes mid-morning or mid-afternoon in summer and with as much skin exposed as feasible at noon in winter, on most days, is likely to be helpful in maintaining adequate levels. (relevant to Australia)
7. High sun exposure is to be avoided as a means to achieve high vitamin D status. The use of sunscreen probably does not prevent the effect of solar radiation on vitamin D synthesis.
8. It is possible that 25(OH) D levels above 75 nmol/L are associated with adverse health outcomes. Vita-min D toxicity (with elevated levels of calcium in blood and urine caused by 25(OH) D levels over 220 nmol/L) can be caused by excess supplementation but not by sunlight (which produces maximum 25(OH) D levels up to between 150–200 nmol/L).
9. Physiological and environmental factors associated with serum 25OHD levels have roles in skin cancer risk.
10. Variable associations are reported between vitamin D levels and basal cell carcinoma (BCC), squamous cell carcinoma, and melanoma incidence.
11. UV radiation contributes to skin carcinogenesis, and vitamin D levels above 75nmol/L may be associated with increased risks for BCC and melanoma.
12. Vitamin D levels in the range of 50–75nmol/L appear to be appropriate for those at risk for skin cancer based on available literature.
